# Alcohol use disorder as a risk factor for violent offending and reoffending in delinquent women with substance use disorders

**DOI:** 10.1007/s00737-023-01316-1

**Published:** 2023-04-26

**Authors:** Juliane Mayer, Judith Streb, Ivonne Steiner, Viviane Wolf, Verena Klein, Manuela Dudeck, Irina Franke

**Affiliations:** 1 Department of Forensic Psychiatry and Psychotherapy, kbo-Isar-Amper-Klinikum Taufkirchen (Vils), Braeuhausstr. 5, 84416 Taufkirchen (Vils), Germany; 2 Department of Forensic Psychiatry and Psychotherapy, Ulm University, Lindenallee 2, 89312 Guenzburg, Germany; 3 LVR-Klinikum Duesseldorf, Bergische Landstr. 2, 40629 Duesseldorf, Germany; 4 Psychiatric Services of Grisons, Loestrasse 200, 7000 Chur, Switzerland

**Keywords:** Violent offending, Women, Alcohol use disorder, Substance use disorder, Abuse

## Abstract

Female gender is generally less associated with aggressive behavior and violent offending than male gender. Therefore, most studies on violence and (re-)offending include only men. However, it is crucial to better understand pathways to female offending in order to enable efficient psychological interventions and risk assessment in women. Well-established risk factors for aggressive behavior include alcohol use disorder (AUD) and other substance use disorders (SUDs). We retrospectively analyzed the association of AUD and other SUDs with violent offending and reoffending in a sample of female offenders (*N* = 334) in a forensic treatment facility. In total, 72% of the patients with an AUD had committed a violent crime leading to admission, whereas only 19% of those with other SUDs had. Over 70% of the participants with AUD had a family history of AUD, and over 83% had experienced physical violence in adulthood. Rates of AUD and other SUDs did not differ regarding aggressive behavior during inpatient treatment, while the risk of reoffending with a violent crime after discharge was nine times higher in patients with an AUD than in those with other SUDs. Our results indicate that AUD is a significant risk factor for violent offending and reoffending in women. A familial background of AUD and a history of physical abuse increase the probability for both AUD and offending, suggesting a possible interaction between (epi-)genetic and environmental factors. The comparable rates of aggression during inpatient treatment in patients with AUD and other SUDs indicate that abstinence is a protective factor for violence.

## Introduction

The risk for aggressive behavior and violent offending is clearly higher in men than in women, and this difference has been explained mainly by different biological, psychological, and social influences, including genetic predispositions, hormonal factors (especially testosterone), gender-specific characteristics and expectations, and differences in conflict-solving strategies (Björkqvist 2018; Padgett & Tremblay 2020). Gender is regarded as one of the best predictors for violent behavior, and female violence is widely acknowledged to be of less salience and severity than male violence; both these findings have resulted in a lack of empirical research on violence risk assessment and management in women (de Vogel & Nicholls 2016).

Some authors also mention that female criminal behavior is harder to quantify and qualify because of underreporting and probably milder punishments (Fernando Rodriguez et al. 2006). Moreover, research on convictions because of aggressive behavior shows that female violence is more often indirect and reactive and occurs mostly within relationships, whereas male violence is characterized mainly by instrumental, antisocial, sexual, and peer motives (Cortoni et al. 2010; de Vogel & de Spa 2019).

Although women account for only a relatively small proportion of prisoners and inpatients in secure forensic treatment settings (de Vogel & Nicholls 2016), the absolute numbers of women in detention have grown in the last two decades (Bartlett & Hollins 2018). Therefore, the question has become more relevant whether traditional criminological theories of offending, which were developed for male populations, can be applied equally to women. The existence of specific factors underlying female criminal behavior has been controversially discussed, and so far, studies seem to favor the view that male and female offenders share most of the psychosocial and especially the biological risk factors for criminal behavior (Brennan et al. 2010; Boisvert et al. 2012).

Despite the above findings, research has also shown that certain risk factors have a stronger effect on violent behavior in women than in men (de Vogel et al. 2016). For example, dysfunctional backgrounds with high rates of disadvantage and victimization histories, including childhood abuse (Putkonen et al. 2011), disruptions in relationships and families (Johansson & Kempf-Leonard 2009), and substance abuse by parents (van der Put et al. 2014), have been shown to affect women more strongly than men. Conversely, a more recent study in 1571 Swiss female prisoners under forensic psychiatric care (Krammer et al. 2018) showed that the prevalence of negative childhood events did not differ between women with or without violent offenses. On the other hand, Salisbury and Van Voorhis (2009) suggested the existence of gender-specific pathways to crime and found that traumatization during childhood does not directly influence criminal behavior. Instead, mental disorders and substance abuse among traumatized women appear to be predictors for later criminal behavior, with the latter functioning as some form of self-medication to reduce trauma-related symptoms (Khantzian 1997).

The link between substance abuse disorders (SUDs) and violent behavior is well established (Whiting et al. 2021). In the case of alcohol use disorder (AUD), evidence from laboratory and empirical research even supports a possible causal role when it comes to violent behavior (Boles & Miotto 2003). Although this was shown to be true for both genders, studies on alcohol consumption and violent behavior with different operationalizations of violence, including laboratory-observed violence (Giancola et al. 2009) and self-reported violence (Bellis et al. 2018), have shown that alcohol use has greater effects on aggressive behavior in men than in women (Duke et al. 2018). However, it remains unclear whether violence is a direct and monocausal effect of alcohol on the central nervous system or whether and to what extent mediating factors, such as personal, social, environmental, and cultural ones, play an additional role (Lipsey et al. 2002; Smyth 2013).

Regarding arrests for violent behavior, Martin and Bryant (2001) explored gender differences in substance use in an American sample of 9242 men and 2594 women in relation to arrests for violent and property offenses. They found that, while intoxication was significantly more likely to precede arrest for a violent offense in both genders, the effect of alcohol intoxication as a predictor of a violent crime was more than three times stronger in the women. Most notably, the likelihood of arrest for a violent crime in the female sample increased more than fivefold in women who were under the influence of alcohol, whereas women abusing cocaine or other drugs were significantly more likely to be arrested for a property offense (Martin & Bryant 2001). Similarly, although both men and women with an AUD were more likely than offenders without an AUD to be rearrested for a violent offense (Castillo & Fiftal Alarid 2011), the role of alcohol abuse in reoffending with violent crimes proved to be more relevant in women (Chang et al. 2015). In summary, women with an AUD seem more at risk for violent offending and reoffending than women with other SUDs.

Although considerably more research has been performed on female violence in the past two decades, with publications reporting gender-specific data among offender populations from different countries around the world, there is still an urgent need for a better understanding of specific risk factors, including different types of SUDs, for violent offending and reoffending in female forensic patients (de Vogel & Nicholls 2016). So far, most evidence has been derived from female prisoners in correctional facilities. However, offenders in forensic treatment facilities are detained under court-ordered treatment because they have a serious mental disorder or SUD. The disorder is directly related to the offense committed, which often diminishes or even voids responsibility for the offense and also increases the risk for reoffending without treatment (Franke et al. 2020).

In Germany, admission to a forensic treatment facility follows a court decision according to Sect. 63 or 64 of the German Criminal Code. Hospitalization according to Sect. 64 requires a diagnosis of AUD or SUD, a high risk of reoffending, and a favorable treatment prognosis; it does not necessarily imply diminished criminal responsibility and has a standard duration of two years. Patients hospitalized according to Sect. 64 are treated on separate wards specialized in SUD treatment. Upon regular discharge from treatment, patients are placed on conditional release and must comply with mandatory treatment orders, such as attending outpatient care, abstaining from substance use, living in supportive facilities, and participating in regular employment.

A diagnosis of AUD or another SUD is less common in women than in men (Schuckit 2011); however, the gender gap is narrowing (Colell et al. 2013). Still, given the relatively small number of female offenders in general and female forensic patients in particular, studies on female forensic patients with SUDs—including their pathways to crime and risk factors for violent offending and reoffending—are scarce and face methodical challenges, such as small sample sizes and short follow-up periods (Krammer et al. 2016). To our knowledge, only three studies have gathered information on women in forensic treatment for SUDs in Germany regarding risk factors for offending and reoffending. Maaß and Schläfke (2016) studied 15 female patients in a forensic treatment facility in northern Germany. They gathered descriptive data on psychopathology, criminal history, family background, and experiences with violence, finding first-hand experience of violence and an unstable family background to be the most important risk factors for criminal behavior. Frey (2019) analyzed data on treatment outcome in 27 female patients in four forensic treatment facilities in Germany and found that 37% of the sample recidivated and that motherhood significantly reduced the probability of reoffending. However, because of the small sample sizes, neither study examined an association of the different types of SUDs, gender-specific risk factors, and violent offending. Franke et al. (2020) reported on patient characteristics and the outcome of forensic treatment for SUDs in Germany and showed that women had a lower level of psychosocial functioning before intake and after discharge than men but did not differ with respect to relapses or rates of reoffending.

This study aimed to gain more insight into the association of different types of SUDs and violent offending and reoffending in female forensic patients with AUD or other SUDs. To further explore female pathways to crime leading up to hospitalization in a forensic treatment facility, we also analyzed family background and victimization histories as often cited gender-specific risk factors for violent offending in relation to the different types of SUDs. The results may have implications for risk assessment and treatment of women with SUDs beyond forensic treatment settings and thus lead to a better understanding of this specific group of forensic patients and ultimately more effectively prevent violent (re)offending.

## Materials and methods

### Sample characteristics

The sample included 418 records of female inpatients at a secure forensic psychiatric hospital in Bavaria (Germany) who received court-ordered treatment for SUDs (according to Sect. 64 of the German Criminal Code) and were discharged between 2001 and 2018. After the first screening, 84 cases had to be excluded because of missing values, so 334 cases were included in the analyses. Patient consent was waived because data were analyzed retrospectively in a way that did not allow identification of individual participants.

The mean age at admission was 33.02 years (range, 17–59 years), the mean duration of inpatient treatment was 23.46 months (range, 1–67 months), and the mean follow-up period was 11 years (median, 2918.74 days; range, 748–6918 days).

### Measures

Based on an extensive literature review on gender-specific risk factors for recidivism, we designed a codebook in collaboration with the Office of Corrections and Rehabilitation, Zurich, Switzerland. The study assessed sociodemographic variables (e.g., age), gender-specific risk variables (e.g., SUD in the family, experiences of physical violence), criminological variables (e.g., index offense), and treatment variables (e.g., physical violence during detention). Data were collected from archived patient records, including official court documents. During treatment, diagnoses were made according to the ICD-10 criteria.

Information about criminal recidivism was obtained from the German Federal Office of Justice, which provided entries on convictions in the Federal Central Criminal Register for September 2020 and, because some data were missing in the response to the initial request, again in February 2021. Two binary measures (i.e., yes/no) were used: general recidivism, which was defined as a conviction for any new offense; and violent recidivism, which was defined as a conviction for an offense involving crimes against persons (e.g., murder, manslaughter, attempted murder, sexual assault, assault, harassment, and robbery). Only convictions committed and sentenced after release were included in the assessment of recidivism.

### Statistical methods

Data were analyzed with IBM SPSS Statistics for Windows Version 27 (IBM Corp., Armonk, NY, USA). For descriptive statistics, absolute and relative frequencies, mean values, standard deviations, and ranges were calculated. Differences in index offenses, comorbidities, gender-specific risk factors, treatment variables, and recidivism between patients with AUD and those with other SUDs were analyzed by Pearson’s chi-square tests and Fisher’s exact test. Furthermore, Kaplan–Meier survival curves were plotted to assess recidivism rates over time. A *p*-value of less than 0.05 was considered to indicate statistical significance.

## Results

### Diagnosis

AUD (ICD-10 F10.2) was the primary diagnosis (i.e., the diagnosis leading to admission) in 16% (*n* = 53) of the participants; another SUD, including opioids (ICD-10 F11.2), cannabinoids (ICD-10 F12.2), sedatives and hypnotics (ICD-10 F13.2), cocaine (ICD-10 F14.2), and other stimulants (ICD-10 F15.2), in 39% (*n* = 131); and multiple SUDs and use of other psychoactive substances (ICD-10 F19.2) in 45% (*n* = 150; see Table 1).

**Table 1:** Frequencies of primary diagnosis and comorbid personality disorders in the total sample (*N* = 334)

	Primary diagnosis	Comorbid personality disorder	
	*n* (%)	Emotionally unstable *n* (%)	Dissocial *n* (%)
Alcohol	53 (16%)	11 (3.2%)	4 (1.2%)
Opioids	74 (22%)	3 (1%)	1 (0.3%)
Cannabinoids	7 (2%)	1 (0.3%)	0
Sedatives or hypnotics	3 (1%)	0	0
Cocaine	5 (1.5%)	0	0
Other stimulants	42 (12.5%)	3 (1%)	1 (0.3%)
Multiple drug use	150 (45%)	22 (6.5%)	11 (3.3%)
Total	334 (100%)	40 (12%)	17 (5%)

In total, 17% (*n* = 57) of the patients were diagnosed with a comorbid personality disorder, i.e., emotionally unstable (ICD-10 F60.3), 12%, *n* = 40, and dissocial (ICD-10 F60.2), 5%, *n* = 17 (see Table 1). The diagnostic groups of the SUDs did not differ with regard to the prevalence of comorbid personality disorders (emotionally unstable, Fisher’s exact test = 10.842, *p* = 0.066; dissocial, Fisher’s exact test = 5.292, *p* = 0.450). The index offense (i.e., the crime leading to admission) was homicide in 5% (*n* = 17), assault in 17% (*n* = 55), robbery in 5% (*n* = 18), violations of the narcotics act in 52% (*n* = 175), and other (non-violent) crimes in 21% (*n* = 69).

### Violence and AUD or other SUDs

In total, 72% of the patients with AUD committed a violent offense (homicide, assault, robbery) that resulted in admission, but this rate was significantly lower (19%) in the group with other SUDs (*χ*^2^ (24) = 169.033; *p* < 0.001).

### Family history of AUD or other SUDs and experience of physical violence

In the whole group, over 70% of patients had a positive family history for AUD, another SUD, or both. In the patients with AUD, a positive family history for AUD was noted in over 70%, and this cell has the largest deviation of the observed value from the expected value (*χ*^*2*^(18) = 51.518; *p* < 0.001; see Table 2).

**Table 2:** Family history of alcohol use disorder and other substance use disorders

	Alcohol and other substance use disorders in the family (i.e., parents, siblings, grandparents, aunts, and uncles)			
	No *n* (%)	Alcohol use disorder *n* (%)	Other substance use disorder *n* (%)	Both types of substance use disorder *n* (%)
Alcohol (*n* = 51)	10 (20%)	36 (71%)*	0	5 (10%)
Opioids (*n* = 70)	28 (40%)	16 (22%)	9 (13%)	17 (24%)
Cannabinoids (*n* = 7)	2 (29%)	3 (43%)	2 (29%)	0
Sedatives or hypnotics (*n* = 3)	2 (67%)	0	0	1 (33%)
Cocaine (*n* = 5)	3 (60%)	1 (20%)	0	1 (20%)
Other stimulants (*n* = 41)	9 (22%)	11 (27%)	11 (27%)	10 (24%)
Multiple drug use (*n* = 148)	40 (27%)	58 (39%)	17 (12%)	33 (22%)
Total (*N* = 325)	94 (29%)	125 (39%)	39 (12%)	67 (21%)

*Cell with the largest standardized residual amount

Patients with AUD have experienced physical violence in adulthood in over 80% of the cases; this cell has the largest deviation of the observed value from the expected value (*χ*^2^(6) = 24.969; *p* < 0.001). With the exception of patients with cannabinoid use disorder as the SUD (0%), rates of experienced physical violence in childhood and adolescence were high in all SUD subgroups (range for individual types of SUD, 28 to 67%). However, we found no significant differences between the different diagnostic groups (*χ*^2^(6) = 7.131; *p* = 0.309; see Table 3).

**Table 3:** Experiences of physical violence in adulthood and childhood/adolescence

	Experiences of physical violence (by family members, partners, friends, or strangers)	
	In adulthood *n* (%)	In childhood/adolescence *n* (%)
Alcohol (*n* = 52)	43 (83%)*	14 (28%)
Opioids (*n* = 72)	29 (40%)	22 (31%)
Cannabinoids (*n* = 7)	3 (43%)	0
Sedatives or hypnotics (*n* = 3)	1 (33%)	2 (67%)
Cocaine (*n* = 4)	1 (25%)	2 (40%)
Other stimulants (*n* = 42)	24 (57%)	13 (31%)
Multiple drug use (*n* = 148)	83 (56%)	57 (39%)
Total (*N* = 325)	184 (56%)	110 (34%)

*Cell with the largest standardized residual amount

### Violence in patients with AUD and other SUDs during treatment

In contrast to the rates of violent offending leading to admission in the AUD group, the rates of institutional violence during hospitalization were generally low (8%, *n* = 28). We found no statistically relevant difference between the patients with AUD and those with another type of SUD (*χ*^2^(6) = 4.093; *p* = 0.664; see Table 4).

**Table 4:** Physical violence during hospitalization

	Physical violence during hospitalization (against objects, fellow patients, or staff) Yes *n* (%)
Alcohol (*n* = 53)	7 (13%)
Opioids (*n* = 74)	4 (5%)
Cannabinoids (*n* = 7)	0
Sedatives or hypnotics (*n* = 3)	0
Cocaine (*n* = 5)	0
Other stimulants (*n* = 42)	3 (7%)
Multiple drug use (*n* = 150)	14 (9%)
Total (*N* = 334)	28 (8%)

### Risk of violent and general reoffending

The rate of violent reoffending was 13% and that of general reoffending 25%. Patients with AUD were significantly more likely than patients with other SUDs or multiple SUDs to reoffend with a violent crime after discharge (*χ*^2^(6) = 21.850; *p* < 0.001; see Table 5).

**Table 5:** Rates of violent and general reoffending

	Violent reoffending *n* (%)	General reoffending *n* (%)
Alcohol (*n* = 53)	14 (26%)*	25 (47%)
Opioids (*n* = 74)	3 (4%)	33 (45%)
Cannabinoids (*n* = 7)	0	0
Sedatives or hypnotics (*n* = 3)	0	3 (100%)
Cocaine (*n* = 5)	0	1 (20%)
Other stimulants (*n* = 42)	1 (2%)	11 (26%)
Multiple drug use (*n* = 150)	24 (16%)	86 (57%)
Total (*N* = 334)	42 (13%)	189 (25%)

*Cell with the largest standardized residual amount

**Fig. 1:**
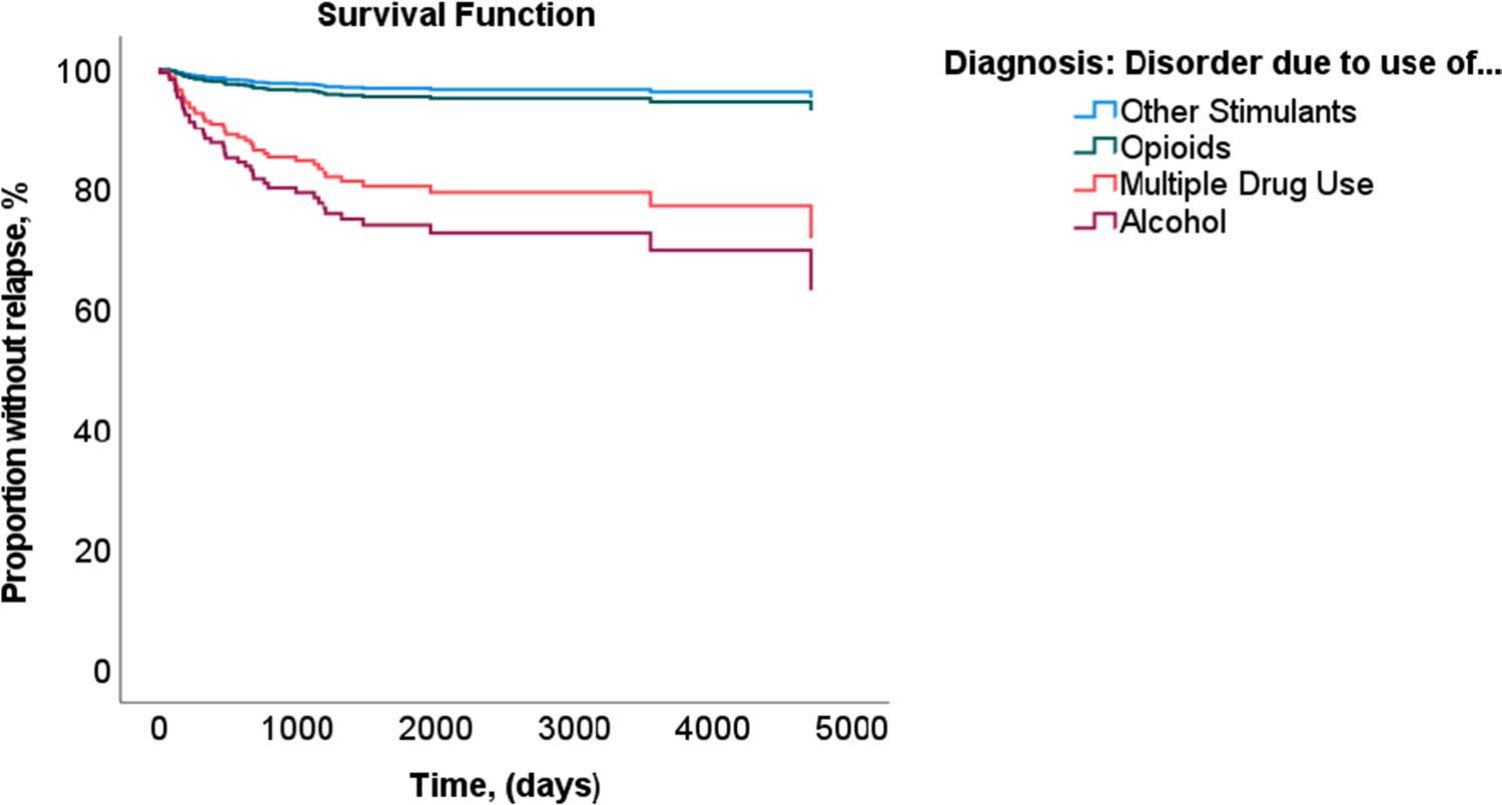
Risk of violent reoffending in female inpatients with alcohol use disorder (*n* = 53) or a substance disorder due to use of opioids (*n* = 74), other stimulants (*n* = 42), or multiple drug use (*n* = 150)

The risk of reoffending with a violent crime was nine times higher in patients with an AUD than in those with other SUDs (hazard ratio [HR] = 9.456; *p* = 0.030). The risk in patients with a multiple drug use disorder (HR = 6.830; *p* = 0.060) or opioid use disorder (HR = 1.451; *p* = 0.747) was not significantly different from that in patients with a disorder due to use of other stimulants. Patients with SUDs due to cannabinoids, sedatives or hypnotics, or cocaine were excluded from the analysis because the sample sizes were too small. The critical time at risk for violent reoffending was approximately 5 years after discharge (see Fig. 1).

**Fig. 2:**
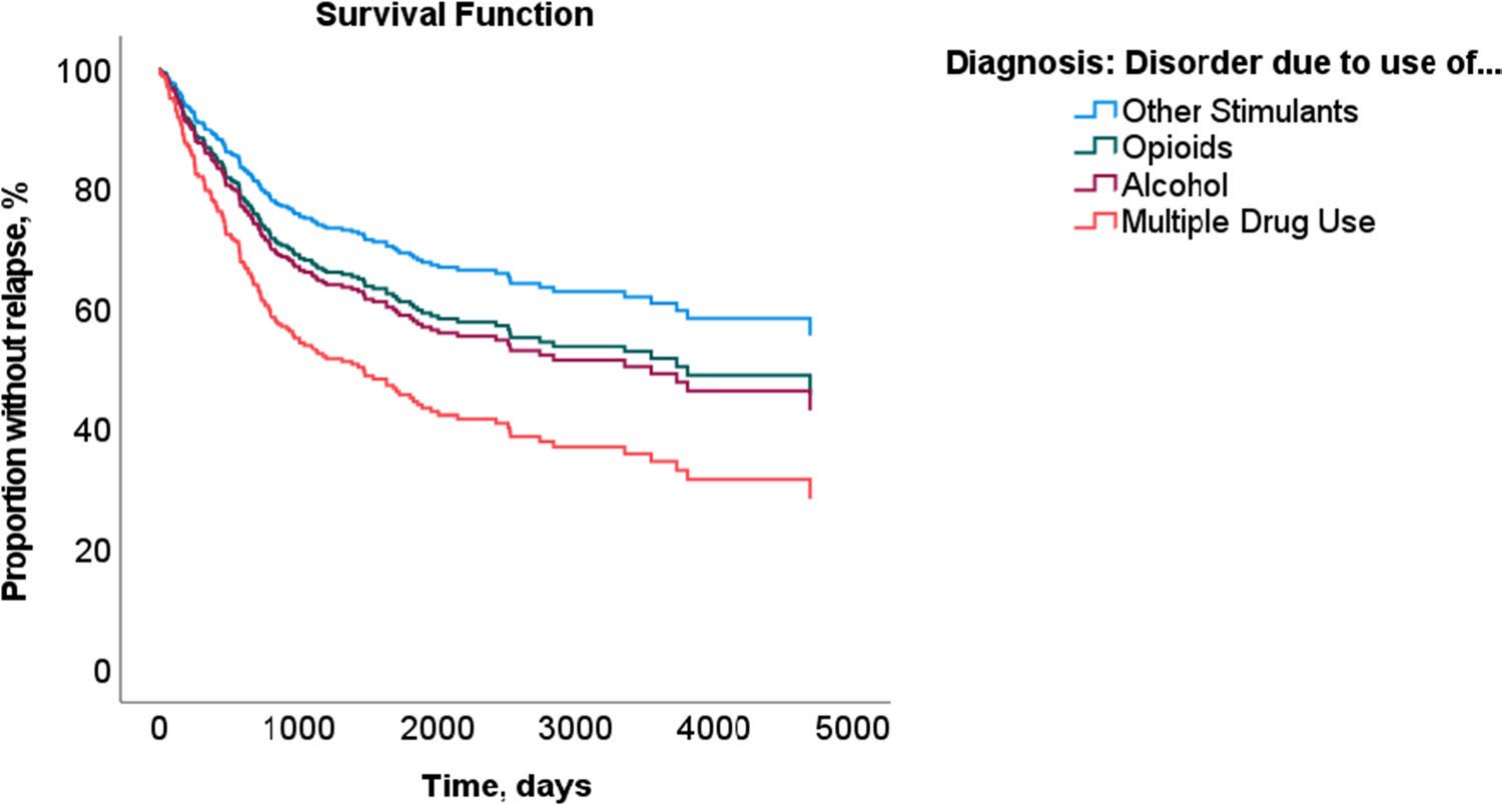
Risk of general reoffending in female inpatients with alcohol use disorder (*n* = 53) or a disorder due to use of opioids (*n* = 74), other stimulants (*n* = 42), or multiple drug use (*n* = 150)

The results concerning the rates for general reoffending were different in that the risk in patients with a disorder due to multiple drug use was twice that in patients with a disorder due to use of another drug (HR = 2.147; *p* = 0.017). Patients with an AUD (HR = 1.442; *p* = 0.316) or opioid use disorder (HR = 1.339; *p* = 0.404) did not differ significantly from those with a disorder due to other stimulants. Patients with a disorder due to use of cannabinoids, sedatives, or cocaine were excluded from the analysis because the respective sample size was too small. The critical time at risk for general reoffending was also approximately 5 years after discharge (see Fig. 2).

In patients with a comorbid emotionally unstable personality disorder, the risk of violent reoffending was twice that in the group without this comorbidity (HR = 2.237; *p* = 0.033), but the risk of general reoffending was not significantly higher (HR = 1.538; *p* = 0.052).

## Discussion

The aim of the present study was to analyze the association of AUD and other SUDs with violent behavior in a female offender population in a forensic treatment setting. We analyzed data from the only forensic treatment facility exclusively for women in Germany. Spanning 18 years, the data represent a substantial body of evidence. Thus, we were able to present a rather large sample size (*N* = 334) and long mean follow-up period (11 years), countering earlier research and methodological problems of studies with this particular group of forensic patients (Krammer et al. 2016).

Our results indicate that AUD is associated significantly more often with violent offenses in delinquent women with problematic substance use than other SUDs. This association was found for both the index offense and the risk of violent reoffending. In contrast, the AUD group had a significantly lower risk of general reoffending than the group with other SUDs. These findings can be explained by the differences in psychophysiological action and effect of the substances: Opiates mute and contain affects of rage and aggression, while stimulants can counteract hyperactivity, emotional instability, and inattentiveness (Khantzian 1997). Alcohol, on the other hand, has shown to have a negative effect on inhibition or impulse control, supporting the hypothesis of a biological link between alcohol and aggression (Boles & Miotto 2003).

Accordingly, the AUD group and the group with other SUDs showed no difference in violent behavior during hospitalization. As substance use during hospitalization is prohibited and carefully monitored, one might interpret this difference as to abstinence being a de facto protective factor for violent behavior. However, the living conditions in secure psychiatric hospitals might also have contributed to this effect because women mostly show violent behavior within close relationships (de Vogel & de Spa 2019), which can hardly be established during hospitalization and are very challenging to maintain.

Another interesting result was that a significantly higher proportion of participants with AUD had a positive family history of AUD and had experienced violence in adulthood. This result mirrors findings on the relevance of additional (epi-)genetic and/or environmental risk factors for both violent offending (Tremblay et al. 2018) and AUD, with heritability and environment each explaining about half of the variance for alcohol dependence (Dick & Bierut 2006). It is also in line with previous research that found higher prevalence rates for interpersonal trauma in women who show violent and general offending behavior (Putkonen et al. 2011; de Vogel et al. 2016; Krammer et al. 2018). The result therefore corresponds with the psychophysiological effect of alcohol in permitting the experience of affection, aggression, and closeness in individuals who are otherwise cut off from their feelings and relationships (Khantzian 1997). Moreover, the fact that we found women with AUD to have less frequently experienced violence in their childhood concurs with findings by Krammer et al. (2018), indicating that the cycle of violence theory according to Widom (1989) is less suitable for explaining violent acts by women with violent childhood experiences.

Still, we found high rates of victimization in all groups of SUDs. This can be explained when considering correlates of addictive behavior and the associated dysregulations of brain reward circuitry. Women in general are more susceptible to the negative reinforcing motivation for substance use than men (Khantzian 1997), i.e., they are more likely to use drugs to regulate internal states of distress. Self-reports from female adolescents also suggest that coping with negative affective states, such as anxiety, depression, low self-esteem, and feelings of isolation, is an important motivator for starting to use alcohol (Whiting et al. 2021). The main neuronal systems involved in gender differences in addiction are dopamine, opioid receptors, and brain-derived neurotrophic factors (Boles & Miotto 2003). In addition, estrogen seems to contribute to an increased sensitivity to drug-associated cues and stress and to affect individual vulnerability and relapse in women (Khantzian 1997). The neurobiological differences in the mechanisms of addiction interact with gender-specific societal and epigenetic effects: The most prominent finding is that women with SUDs more often have a history of interpersonal trauma than men (Giancola et al. 2009). Thus, a possible implication for treatment is that female offenders with AUD need a careful diagnostic assessment of trauma, especially because symptoms of SUDs and trauma may overlap but require different treatment strategies. In consequence, female forensic patients face special challenges, such as coping with often severe and violent offenses and, at the same time, complex personal and psychiatric problems (de Vogel et al. 2016). An implication for the treatment of female general psychiatry patients with AUD would be to thoroughly evaluate for any interpersonal violence experience and to provide tools for conflict solving, help-seeking, and domestic violence counseling, in order to prevent violent offending.

This study also adds important information about the risk of reoffending in women in general. Over the 11-year follow-up period, we found a rate of 25% for general reoffending and 13% for violent offending. The most vulnerable time for reoffending seems to be within the first 5 years after discharge, which underlines the importance of sufficient aftercare and supervision. Overall, our results are in line with previous findings (Boduszek et al. 2014; Chang et al. 2015; Martin & Bryant 2001) that AUD is an important risk factor for violent offending and reoffending in delinquent women with SUDs and that women with AUD should be closely monitored after discharge from secure settings.

The findings of the present study have to be interpreted with several limitations in mind. Most importantly, the lack of a control group limits the interpretation of our results, as we did not assess non-delinquent women with AUD and other SUDs (e.g., general psychiatry patients), nor did we sample female offenders without SUDs (e.g., in a correctional setting). Furthermore, the study was conducted retrospectively and used only information from files. As a result, missing data could not be determined retrospectively and we could neither verify diagnostic and historic information, nor follow-up for relapses and unregistered violent behavior. Because we do not have any detailed information on the type (e.g., intimate partner violence) or context (e.g., whether women were both victims and perpetrators; whether substances were used) of reoffending after discharge, our results do not allow conclusions to be drawn about the causalities of violence and AUD. Future research could therefore include appropriate control groups and focus more closely on the effectiveness of forensic treatment in terms of abstinence and stability of the women after discharge as well as the nature of the offenses to draw more nuanced conclusions.

Moreover, we obtained only data from a single hospital, thereby limiting general transferability and the number of cases. On the other hand, collecting data from a single facility can also represent a methodological advantage because of the homogeneity of data acquisition and exclusion to a great extent of other influencing variables, such as regional or national differences in data recording or file keeping.

## Data Availability

The datasets generated for this study are available on request to the corresponding author.
